# Course of neurological soft signs in first-episode schizophrenia: Relationship with negative symptoms and cognitive performances

**DOI:** 10.1038/srep11053

**Published:** 2015-06-08

**Authors:** Raymond C. K. Chan, Fu-lei Geng, Simon S. Y. Lui, Ya Wang, Karen K. Y. Ho, Karen S. Y. Hung, Raquel E. Gur, Ruben C. Gur, Eric F. C. Cheung

**Affiliations:** 1 Neuropsychology and Applied Cognitive Neuroscience Laboratory, Key Laboratory of Mental Health, Institute of Psychology, Chinese Academy of Sciences, Beijing, China; 2 University of Chinese Academy of Sciences, Beijing, China; 3 Castle Peak Hospital, Hong Kong Special Administration Region, China; 4 Department of Psychiatry, Perelman School of Medicine, University of Pennsylvania, and the Philadelphia Veterans Administration Medical Center, Philadelphia, United States of America

## Abstract

This prospective study examined the course of neurological soft signs (NSS) in patients with first-episode schizophrenia and its relationship with negative symptoms and cognitive functions. One hundred and forty-five patients with first-episode schizophrenia were recruited, 29 were classified as having prominent negative symptoms. NSS and neuropsychological measures were administered to all patients and 62 healthy controls at baseline. Patients were then followed-up prospectively at six-month intervals for up to a year. Patients with prominent negative symptoms exhibited significantly more motor coordination signs and total NSS than patients without prominent negative symptoms. Patients with prominent negative symptoms performed worse than patients without negative symptoms in working memory functions but not other fronto-parietal or fronto-temporal functions. Linear growth model for binary data showed that the prominent negative symptoms were stable over time. Despite general improvement in NSS and neuropsychological functions, the prominent negative symptoms group still exhibited poorer motor coordination and higher levels of NSS, as well as poorer working memory than patients without prominent negative symptoms. Two distinct subtypes of first-episode patients could be distinguished by NSS and prominent negative symptoms.

Neurological soft signs (NSS) have been considered among the target features^1^ and a potential endophenotype^2^ for schizophrenia. The investigation of NSS has provided important information in the search for the aetiology of the illness^3^,^4^,^5^. A recent meta-analysis showed that NSS is prevalent in patients with first-episode schizophrenia (Cohen’s d ranges from 0.68 to 1.53) and chronic schizophrenia (Cohen’s d ranges from 0.87 to 1.61), suggesting that the presence of NSS is not confounded by medication side effects and is stable across the different stages of the illness^6^,^7^.

Recent imaging findings have challenged the traditional view that NSS are “non-localizing signs”. A recent meta-analysis^8^ on NSS in schizophrenia suggests that NSS are associated with specific brain structural and functional connectivity changes, mainly involving the cerebello-thalamo-prefrontal network proposed by Andreasen *et al.*^9^. These regions and neural connections overlap with neural substrates thought to be related to the deficit syndrome of schizophrenia or persistent negative symptoms^10^,^11^,^12^.

On the other hand, negative symptoms account for much of the long-term morbidity and poor functional outcome of patients with schizophrenia^13^, and treatment of negative symptoms has remained unsatisfactory despite the advent of second generation antipsychotics^14^. Traditionally there are two approaches for defining negative symptoms in schizophrenia, namely the deficit syndrome^15^ and persistent negative symptoms^16^. The prevalence of the deficit syndrome was estimated to be about 15% in first-episode schizophrenia patients and about 25–30% in patients with chronic schizophrenia^17^; whereas the prevalence of persistent negative symptoms varied from 13.2% to 27% in first-episode schizophrenia according to various operational definitions^18^. Schizophrenia patients with the deficit syndrome were found to have impairment in fronto-parietal lobe functions, but not temporal lobe functions such as verbal and visual explicit memory^10^,^11^,^12^,^19^; whereas patients with persistent negative symptoms were found to have abnormalities in both frontal and temporal lobe structures and functions^18^. It has been suggested that the diagnosis of deficit syndrome schizophrenia may be difficult, especially in first-episode patients^20^, even though the deficit/non-deficit categorization appears to be stable^21^.

Patients with deficit syndrome schizophrenia exhibit significantly higher prevalence of deficits in motor coordination, sensory integration and sequencing of complex motor acts signs compared to those without the deficit syndrome^19^. Galderisi *et al.*^19^ further showed that after controlling for the influence of extrapyramidal symptoms, the deficit/non-deficit category was the only clinical variable entering the regression equation on the sequencing of complex motor acts factor, while the negative symptom dimension was associated with the sensory integration factor. Mittal *et al.*^22^ also showed that ultra-high risk individuals exhibit a decrease in factional anisotropy value in the superior cerebellar peduncle pathway on follow-up after 12 months as compared to healthy controls, although there was no significant difference in the integrity of this pathway at baseline. Taken together, these findings suggest that negative symptoms share common neural substrates with NSS in schizophrenia, especially in the cerebello-thalamo-prefrontal connection. However, Galderisi *et al.*’s study was limited by a relatively small sample size, patients with a long duration of illness and a cross-sectional design. It is also unclear whether the findings were confounded by chronicity of illness or antipsychotic medication effect.

Despite the substantial number of studies on NSS in the past decades, several issues remain unresolved. One of the most important criteria of a potential endophenotype is its stability across the different stages of the illness. The aforementioned studies were all limited by their cross-sectional design. It is still not clear how the prevalence and nature of NSS evolve and change over the course of illness. This gap in knowledge highlights the need for a prospective longitudinal study. To date, nine studies had specifically examined the prevalence of NSS in patients with first-episode schizophrenia using different NSS scales such as the Neurological Evaluation Scale (NES)^23^, the Cambridge Neurological Inventory (CNI)^24^, and the Heidelberg Scale^25^. However, most of them were limited by small sample sizes^26^,^27^,^28^,^29^, a short follow-up period^30^,^31^ or two-time points follow-up^26^,^28^,^29^, the absence of control comparison^29^,^32^ and a lack of neurocognitive function assessments^30^,^32^,^33^,^34^ (see Table 1).

Another unresolved issue is the relationship between NSS and the symptomatology of schizophrenia. It is especially unclear how NSS associate with negative symptoms throughout the course of the illness. Chen *et al.*^33^ have demonstrated that the association between NSS and negative symptoms was not apparent at least one year after the onset of illness and the association tended to increase up to the third year of the follow-up period. However, they only administered the motor coordination subscale of the Cambridge Neurological Inventory (CNI)^24^, which forms only part of the construct of NSS. Prikryl *et al.*^29^ examined patients with and without remission four years after the onset of schizophrenia and found that there was a significant association between NES scores and negative symptoms in patients with first-episode schizophrenia at baseline and four years later. However, their sample was biased towards male participants. It is not clear whether patients with first-episode schizophrenia with prominent negative symptoms would demonstrate a strong association with NSS as compared to patients without prominent negative symptoms, and whether these two subgroups would follow different pathways of evolution over the course of the illness.

The purpose of the present study was to examine the course of NSS in patients with first-episode schizophrenia and its relationship with negative symptoms and cognitive functions. In particular, we attempted to track and compare the changes in NSS between patients with and without prominent negative symptoms within one year of illness onset. We hypothesized that: (1) patients with prominent negative symptoms would exhibit significantly higher prevalence of NSS than patients without prominent negative symptoms; (2) such a pattern would persist within the first year of illness onset at baseline, 6-month, and 12-month intervals; (3) fronto-parietal dysfunctions (working memory) but not fronto-temporal dysfunctions (verbal and visual explicit memory) would be more impaired in patients with prominent negative symptoms than patients without prominent negative symptoms.

## Results

### Attrition analysis

A total of 36 patients dropped out from the study (attrition rate = 24.8%). Among the patients who dropped out, one committed suicide, two did not return for follow-up, and the remaining 33 patients refused further assessments, even though they continued to attend clinical appointments. A comparison of patients who completed the assessments and those who dropped out showed that there were no significant differences in age ((*t* (143) = 0.17, *P* = 0.863), (X^2^(1) = 0.52, *P* = 0.472), education (*t* (143) = 0.19, *P* = 0.849), handedness (X^2^(1) = 0.69, *P* = 0.406), and IQ estimates (*t* (143) = 1.11, *P* = 0.271). The two groups did not differ in the proportion of patients with and without prominent negative symptoms (X^2^(1) = 0.15, *P* = 0.701). The two groups also did not differ in antipsychotic medication dosage (t (1280 = 1.957, *P* = 0.053). However, the dropped-out patients had significantly lower levels of positive symptoms (*t* (143) = 3.42, *P* = 0.001), general psychopathology subscale scores (*t* (143) = 2.19, *P* = 0.030) and total score on the PANSS (*t* (143) = 2.20, *P* = 0.030), suggesting that those who refused to participate were less symptomatic.

### Cross-sectional analysis

ANOVAs on the IQ estimates indicated that both patients with and without prominent negative symptoms had significantly lower IQ estimates than healthy controls ((*F* (2, 143) = 13.67, *P* < 0.001), and both patient groups had significantly more male participants than the healthy control group (*X*^*2*^ (2) = 6.91, *P* = 0.032) (Table 2). The three groups did not differ in handedness, age, and years of education. An examination of clinical symptoms showed that patients with prominent negative symptoms had, as expected, higher negative symptoms subscale score (*t* (143) = 15.19, *P* < 0.001), general psychopathology subscale score (*t* (143) = 3.28, *P* = 0.001) and total PANSS score (*t* (143) = 6.14, *P* < 0.001). The two groups did not differ in positive symptoms subscale score (*t* (143) = 1.28, *P* = 0.203). There were also no significant differences between the patient groups in scores on the Simpson-Angus Scale^35^ (*t* (143) = 1.55, *P* = 0.132), the Barnes Akathisia^36^ Scale (*t* (143) = 0.04, *P* = 0.972) and the Abnormal Involuntary Movement Scale^37^ (*t* (143) = 0.78, *P* = 0.438).

Table 3 summarizes the comparison of NSS and neuropsychological functions between patients with and without prominent negative symptoms, and healthy controls at baseline. An examination of the level of NSS showed that there were significant differences between the three groups in motor coordination (*F*(2, 141) = 13.47 *P* < 0.001), sensory integration (*F*(2, 141) = 19.02, *P* < 0.001) and total NSS score (*F*(2, 141) = 19.02, *P* < 0.001). Independent t tests showed that patients with and without prominent negative symptoms had significantly more total and subscale scores of NSS than healthy controls. Moreover, patients with prominent negative symptoms had significantly more motor coordination impairment (*t*(143) = 2.64, P = 0.009) and total NSS (*t* (143) = 2.36, *P* = 0.019) than patients without prominent negative symptoms.

MANCOVA controlling for gender and IQ showed significant differences in neuropsychological performance between patients with and without prominent negative symptoms and healthy controls. Both patient groups performed worse than healthy controls on neuropsychological tests. Independent t tests further indicated that patients with prominent negative symptoms performed worse than patients without negative symptoms in logical memory (*t* (143) = 2.01, *P* = 0.047), delay logical memory (*t* (143) = 2.35, *P* = 0.020), letter number span correct items (*t* (143) = 2.64, *P* = 0.009) and letter number span longest items (*t* (143) = 2.45, *P* = 0.015).

### Longitudinal analysis

The prevalence of prominent negative symptoms in the persistent negative symptoms group was 20.0%, 17.8%, 15.6% at time 1, time 2 and time 3, respectively. Linear growth model for binary data analysis showed that the level of prominent negative symptoms was stable over time (*F* (1,128) = 1.24, *P* = 0.27 for slope).

**Figure 1:**
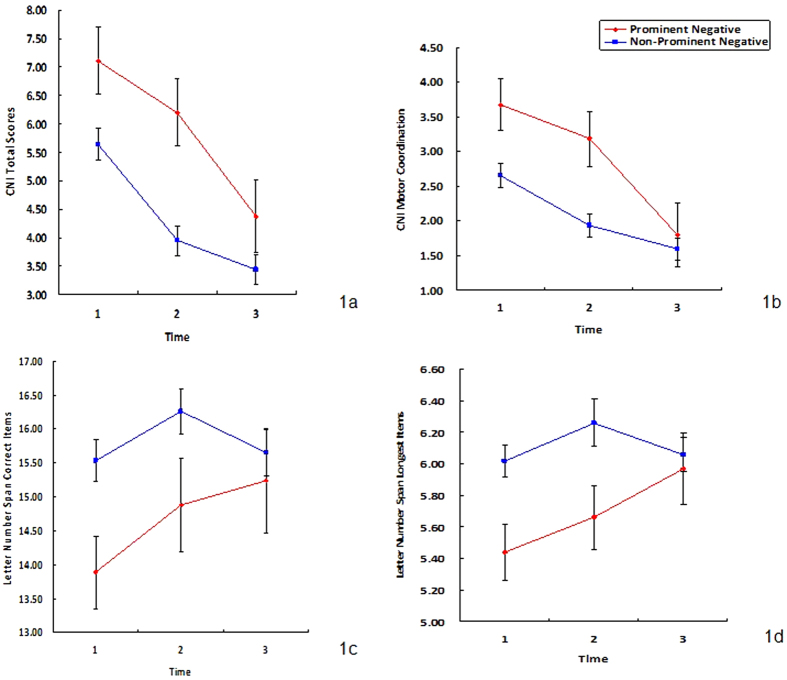
Trajectory measures showing the significant differences found between patients with and without prominent negative symptoms. (1**a**) CNI total score on NSS (F(1, 109) = 4.72, *P* = 0.034) (1**b**) CNI motor coordination score (F(1, 109) = 7.22, *P* = 0.009); (1**c**) LNS total correct score (F(1, 109) = 7.88, *P* = 0.007); (1**d**) LNS longest correct item (F(1, 109) = 5.33, *P* = 0.024).

Table 4 shows the trajectories of NSS, clinical symptoms and neuropsychological performances over the three time points. There was a decreasing trend in NSS (F(1,127) = 41.16, *P* < 0.001), positive symptoms (F(1, 127) = 133.78, *P* < 0.001), general psychopathological symptoms (F(1, 127) = 113.71, *P* < 0.001), and WCST perseverative errors (F(1, 127) = 28.81, *P* < 0.001) in both patient groups. On the other hand, logical memory (F(1, 127) = 36.68, *P* < 0.001), delayed logical memory (F(1, 127) = 56.61, *P* < 0.001) and WCST category (F (1, 127) = 16.19, *P* < 0.001) improved for all patients. Despite this general improvement, patients with prominent negative symptoms still had significantly higher scores indicating greater impairment in motor coordination (F(1,109) = 7.22, *P* = 0.009) and total NSS scores (F(1, 109) = 4.72, *P* = 0.034), as well as poorer performance on the LN test (both correct response and longest items) than patients without prominent negative symptoms (Fig. 1). Moreover, the correlation between NSS and negative symptoms increased gradually across the three time points: 0.188, 0.525 and 0.574, respectively. The correlation coefficient between motor coordination subscale score and negative symptoms was 0.196, 0.608 and 0.402, respectively. Similarly, the correlation coefficient between the sensory integration subscale score and negative symptoms for the three time points was 0.082, 0.187 and 0.566, respectively; and the correlation coefficient between the disinhibition subscale score and negative symptoms was 0.101, 0.165 and 0.256 respectively. There was no significant time and group interaction on the entire test in the study.

## Discussion

The present findings generally confirm results from previous studies examining the prevalence of NSS in first-episode schizophrenia^24^,^26^,^27^,^28^,^29^,^30^. In particular, our findings show that schizophrenia patients with prominent negative symptoms exhibit significantly higher levels of NSS, mainly in the form of motor coordination signs than patients without prominent negative symptoms^19^. These differences were not confounded by IQ and medication side effects. An examination of the NSS abnormalities indicates that these differences were mainly due to differences in the level of motor coordination signs. Motor coordination signs have been associated with frontal lobe connections such as the cortico-cerebellar-thalamic-cortical network^9^ and the cerebello-thalamo-prefrontal network^38^. Our findings highlight the overlap of neural substrates associated with NSS and negative symptoms in schizophrenia^39^.

For neuropsychological performances, when we compared the two patient groups at baseline, we found that patients with prominent negative symptoms did not perform significantly worse than patients without prominent negative symptoms in most of the fronto-parietal and temporal lobe functions, although these two groups both performed significantly worse than healthy controls. Working memory, a fronto-parietal function measured by the LNS test, was the only significant difference between the two patients groups. The linear growth model analysis indicated that patients with prominent negative symptoms continued to show significant differences in working memory as well as NSS (mainly motor coordination signs) from patients without prominent negative symptoms. Unlike patients with the deficit syndrome who show clear and consistent differential frontal-parietal dysfunctions^11^,^19^,^39^, our findings suggest that patients with prominent negative symptoms might exhibit subtle fronto-parietal dysfunction in the early phase of the illness. The patients in our sample were all suffering from their first-episode of schizophrenia and it is possible that they may have a relatively intact fronto-parietal network than patients in the more chronic stage of the illness. The difference in fronto-parietal lobe functions may only manifest in subtle ways in the early stage of the illness that may not be easily detected using behavioural tests. As the illness progresses, such a dysfunction may become more apparent.

In addition, we also tracked the changes of NSS in patients with schizophrenia since illness onset in the first year. Our data showed distinct trajectories of NSS development over the first year of illness, with patients with prominent negative symptoms exhibiting significantly more NSS than patients without negative symptoms. These findings are consistent with our previous three-year follow-up study of a large sample of first-episode schizophrenia patients^33^. In our previous study, it was found that the prevalence of NSS in patients was stable over the first three years after illness onset, and the association of NSS with negative symptoms progressively increased over this time period. However, only motor coordination signs were assessed in that study. Prikryl *et al.*^29^ also found a significant correlation between neurological signs and negative symptoms in patients with first-episode schizophrenia; but they did not specifically track the differential developments of NSS in patients with and without prominent negative symptoms. Moreover, their findings were limited to male patients.

However, it should be noted that the rate of patients with prominent negative symptoms gradually decreased over time in our study. This gradual decrease in the number of patients with prominent negative symptoms might reflect a “regression to the mean” phenomenon in the measurement. Theoretically, it would be more worthwhile to examine the differences in NSS in patients with and without “persistent” negative symptoms, i.e., those in which negative symptoms persist over six or 12 months. However, the number of patients with persistent negative symptoms in the present first-episode schizophrenia sample was so small that it was not feasible to make any meaningful statistical comparisons and interpretation. Further study recruiting a larger sample of first episode patients with persistent negative symptoms is warranted. Moreover, further clarification of the relationships between negative symptoms and NSS should also be examined in future studies. To the best of our knowledge, the present study is the first to specifically track the evolution of a comprehensive range of NSS (motor coordination, sensory integration, and disinhibition) in patients with first-episode schizophrenia with and without prominent negative symptoms.

This study has several limitations. Firstly, the assessing clinicians were aware of the diagnosis and subject status of the participants when they carried out the assessments. However, neuropsychological examination was conducted separately by research assistants. Moreover, the clinicians who rated the NSS and the research assistants who administered the neuropsychological tests were not informed whether the patients were classified as with or without prominent negative symptoms, nor were they aware of our hypotheses concerning neurological signs in schizophrenia. Secondly, the follow-up period of the developmental trajectories of NSS in these two patient groups was short. Notably, participants who dropped out were less ill than those who remained in the study and had lower scores for positive symptoms. Thirdly, given that the present study did not adopt a population-based study design, the prevalence rate of prominent negative symptoms observed in patients with first-episode schizophrenia should be considered as preliminary in nature. Future study adopting a population-based design should be carried out to cross-validate the present findings. Finally, we only used behavioural and clinical measures to capture the neurological and neuropsychological functions. The differences in fronto-parietal functions between patients with first-episode schizophrenia with and without prominent negative symptoms may be so subtle that behavioural measures are not sensitive enough to detect the difference. Moreover, we only adopted several tests to capture “fronto-parietal” and “fronto-temporal” functions. It should be noted that these functions may not be fully captured by a single test and not all tests are localized to a particular brain region. Future study should adopt both structural and functional connectivity measures to capture any trajectory differences between these two distinct groups of patients.

Notwithstanding these limitations, we demonstrated the presence of both NSS and negative symptoms in patients with first-episode schizophrenia. The estimated prevalence of prominent negative symptoms was 20% in our sample and these patients exhibited high prevalence of NSS compared to both patients without negative symptoms and healthy controls. However, the finding that neuropsychological tests showed limited difference between these two distinct groups suggests that NSS may be more sensitive in detecting the underlying clinical and neurological manifestations of schizophrenia. Substantial evidence suggests that clinical features and neurological signs share many similarities, or may even be equivalent^40^ in characterizing the different stages of the schizophrenic illness both behaviourally^3^,^7^,^26^ and morphologically^4^,^22^,^41^. Our findings are consistent with the recent reformulation of schizophrenia as a neurodevelopmental illness characterized by different stages with specific markers for early detection and intervention^42^. The ease of assessment and sensitivity of NSS lends itself as a potentially useful tool for early detection and identification of schizophrenia. A larger scale longitudinal study for a longer period of follow-up could further examine the stability and developmental trajectories of NSS as well as its relationship with prognosis and functional outcome in schizophrenia. Trajectory-based measures integrating neuropsychological functions and structural and functional imaging data may also facilitate the understanding of the pathophysiology of the illness.

## Method

### Subjects

First-episode schizophrenia patients were recruited from the joint research-based first-episode schizophrenia programme between Castle Peak Hospital of Hong Kong and the Key Laboratory of Mental Health, Institute of Psychology, the Chinese Academy of Sciences in Beijing^43^. The programme aims to investigate a number of potential endophenotypic markers using a family study approach. In addition to clinical assessment (phenotyping), detailed endophenotype measurements and DNA collection of both recruited patients and their first-degree relatives are part of the study paradigm. A number of neurocognitive functions thought to have high translational potential have been chosen as potential endophenotypes in this study while the inclusion of proteomics in this project may provide further opportunity to investigate the effect of genotype on psychosis^43^.

The joint programme is based at Castle Peak Hospital and is the research component of a clinical programme in early intervention service for psychosis in Hong Kong. Since 2001, this clinical programme has been the only publicly-funded psychiatric unit covering inpatient, outpatient, and community services for people with first-episode schizophrenia in a local community (Tuen Mun, Yuen Long, Tin Shui Wai) with a population of around one million, constituting 15% of the entire population in Hong Kong. Because of the scarcity of private psychiatric services in the local community, this clinical programme is expected to cover almost all first-episode patients in the catchment area. Beginning in 2011, 145 patients with first-episode schizophrenia were recruited for the present study. The diagnosis of DSM-IV^10^ schizophrenia was ascertained using a “best-estimate” approach based on structured clinical interviews and medical record reviews. Exclusion criteria included (1) a history of substance abuse in the past six months, (2) a history of electroconvulsive therapy in the past six months, (3) a history of neurological disorder, (4) a history of head injury with loss of consciousness for more than 30 minutes, and (5) mental retardation.

At baseline, 123 patients were receiving second-generation antipsychotics (SGA) and eight patients received first-generation antipsychotics (FGA), while 14 patients were un-medicated. Because SGAs were used in the majority of patients, the percentage of maximum BNF (British National Formulary) recommended dose instead of chlorpromazine equivalence was used. The mean dose of antipsychotics was 46.6% of the maximum BNF-recommended dose (SD = 32.1%). Among the 130 patients who were receiving antipsychotics, 32 of them also received anticholinergics (benzhexol, daily dose ranged from 2–8 mg) and two patients received benzodiazepines (lorazepam, daily dose ranged from 1–1.5 mg). Based on medical record review, the mean duration of untreated psychosis and the mean duration of illness were 9.67 months (SD = 16.30 months) and 14.28 months (SD = 17.45 months) respectively. At baseline, among the 145 patients, 74 had received in-patient care.

These patients were then classified into subgroups with and without prominent negative symptoms based on scores on the Positive and Negative Syndrome Scale (PANSS)^44^. We adopted the recommended clinical trial operational classification of patients with prominent negative symptoms proposed by Rainbowitz *et al.*^45^. These criteria include: (1) Baseline score >4 (moderate) on at least three, or >5 (moderately severe) on at least two negative PANSS subscale items^46^,^47^; or (2) PANSS negative subscale: score >3 on item 1 (blunted affect) and at least one third of the items with a score >3 and a maximum of two items with a score >3 from the positive subscale^48^. Based on these criteria, a total of 29 patients were classified as having prominent negative symptoms, while 116 patients were classified as without prominent negative symptoms.

Another 62 healthy controls were identified and recruited from the community. They were recruited from youth centres in the neighbouring community and among the supporting staff of the hospital. They were screened by a semi-structured interview conducted by qualified psychiatrists working in the clinical programme. None of them had any family history of psychiatric illness, or suffered from a neurological illness or alcohol/drug dependence. Intellectual functioning was estimated by the short form of the Chinese version of the Wechsler Adult Intelligence Scale-Revised (WAIS-R)^49^. This method of prorating has previously been used in estimating intellectual functioning in schizophrenia^50^,^51^.

### Neurological soft signs examination

NSS were assessed by the soft signs subscales of the Cambridge Neurological Inventory (CNI)^24^, which has been widely used in patients with schizophrenia in different stages of the illness^6^,^33^,^52^,^53^,^54^. The details of the subscales have been described elsewhere^52^. In brief, there were 25 items in the subscales to capture motor coordination (e.g., fist-edge-palm, finger opposition), sensory integration (e.g., left/right orientation, finger agnosia), and disinhibition (e.g., saccade head movement, go/no go response). Each item was rated on a presence (1) or absence (0) scale. Each item score was summed up to a subscale score for motor coordination, sensory integration, disinhibition, and a total score of NSS. A higher score indicates a higher level of NSS. In this study, NSS were assessed by three qualified psychiatrists (SSYL KKYH KSYH) trained with NSS assessment according to the manual of the CNI. Intra-class correlation coefficients between the raters ranged from 0.85 to 0.91.

### Cognitive function assessment

Fronto-parietal function was assessed by the Letter-Number Span (LNS) test^55^, the modified Wisconsin Card Sorting Test (WCST)^56^ and the verbal fluency test^57^. Fronto-temporal function was assessed by the logical memory and the visual reproduction subtests of the Chinese Wechsler Memory Scale^58^,^59^.

### Procedures

Clinical diagnoses and clinical ratings on negative symptoms were conducted by experienced psychiatrists. Independent ratings on NSS and cognitive tests were administered by trained research assistants who were not aware of the clinical rating and classification of patients with and without prominent negative symptoms. Medication side effects were assessed in patients with schizophrenia using the Simpson-Angus Scale^35^, the Barnes Akathisia^36^ Scale, and the Abnormal Involuntary Movement Scale^37^. The study was approved by the Ethics Committee of the New Territories West Cluster of the Hospital Authority of Hong Kong and the Institute of Psychology, the Chinese Academy of Sciences in Beijing. Written informed consent was obtained from all participants before the administration of all measures. All patients were assessed at baseline, six months, and 12 months, whereas the healthy controls only received baseline assessment.

### Statistical Methods

#### Attrition analysis

In order to compare demographic and neuropsychological variables at baseline between participants who completed all the three assessments and those who dropped out, independent t tests for continuous variables and chi square test for categorical variables were used.

#### Cross-sectional analysis

Chi square tests were used to compare categorical variables (gender and handedness) between the three groups. ANOVAs were used to compare continuous variables (age, education, and IQ estimates) between the three groups. Independent t tests were used to compare clinical symptoms between patients with and without prominent negative symptoms. MANCOVA was used to compare NSS and neuropsychological function performances with gender and IQ as covariates.

#### Longitudinal analysis

Linear growth model for a binary outcome was used to examine the stability of prominent negative symptoms. To compare changes of NSS and neuropsychological variables over time between patients with and without prominent negative symptoms, random growth curve models were performed using PROC MIXED in SAS version 9.0 (SAS Institute Inc; Cary, North Carolina). Gender, group (prominent negative and non- prominent negative), time, and group x time interaction were included as fixed effects and intercept and time as random effect. In these models, compound symmetry covariance structure for repeated subject measures and maximum likelihood were set.

## Additional Information

**How to cite this article**: Chan, R. C. K. *et al.* Course of neurological soft signs in first-episode schizophrenia: Relationship with negative symptoms and cognitive performances. *Sci. Rep.* **5**, 11053; doi: 10.1038/srep11053 (2015).

## Figures and Tables

**Table 1:** Follow-up studies of neurological soft signs in patients with first-episode schizophrenia

**Studies**	**Patients sample**	**Follow-up Period**	**Healthy controls**	**NSS Examination**	**Main Findings**	**Limitations**
Madsen *et al.*, 1999	Thirty-four patients with first-episode schizophrenia and 29 patients with other psychotic disorders were recruited.	Only 18 patients were followed up at 5 years later.	Twenty healthy controls were recruited but only 10 being followed up.	SNE	Patients showed significantly higher prevalence of NSS than healthy controls at baseline assessment. Such a difference increased 5 years later, especially for signs capturing frontal, cortiospoinal and temporo-parietal functions.	The SNE is not a standardized and well-validated scale for the evaluation of NSS in schizophrenia population.The patients and controls samples were very small though the follow-up period was 5 years. There was only 2 time-point assessment that could not reflect general longitudinal changes of NSS in patients with schizophrenia. The association between NSS and negative symptoms was not examined
Whitty *et al.*, 2003	Ninety-seven patients with first-episode schizophrenia and schizophreniform disorders were recruited.	Patients were followed up at 6 months.	Seventy-three healthy controls were recruited and followed up.	NES	Patients showed significant reduction in NSS at the follow-up assessment suggesting that NSS manifested state-like characteristics that varied with clinical course.	The CNE was not a well-validated scale for NSS assessment. The items included in the NES consist of both “hard” and “soft” signs. Despite a reasonable large sample for both patients with first-episode schizophrenia and healthy controls, the follow-up period was limited to a 2 time points at 6-month interval. The association between NSS and negative symptoms was not examined.
Bachmann *et al.*, 2005	Thirty-nine patients with first-episode schizophrenia spectrum disorders	Patients were followed up at 14 months	Twenty-two healthy controls were recruited and followed up at 14 months.	Heidelberg Scale	Patients demonstrated significantly higher NSS prevalence than controls at baseline and follow-up. However, the prevalence of NSS remained stable in patients over the two time points.	The items included in the Heidelberg scale consist of both “hard” and “soft” signs. Relative small samples for both patients and controls and the follow-up period was limited to a 2 time points. The association between NSS and negative symptoms was not examined.
Chen *et al.*, 2005	One hundred and thirty-eight patients with first-episode schizophrenia, and schizophreniform, and schizoaffective disorder were recruited.	Only 93 patients were followed up at 3 years	Sixty-eight healthy controls were assessed at baseline.	Only motor coordination subscale of the CNI	Patients with medication-naïve schizophrenia demonstrated significantly higher prevalence of NSS than healthy controls, and the prevalence of NSS persisted over the 3 years since onset of schizophrenia. The association between NSS and negative symptoms increased gradually from the first year after the initial episode.	The findings were limited to the motor coordination signs. The changes of other signs such as sensory integration and disinhibition were not clear. No additional neurocognitive function assessment was obtained from this study.
Prikryl *et al.*, 2007	Ninety-two male patients with first-episode schizophrenia were recruited.	Patients were followed up at 1 year.	None	NES	Patients with remission demonstrated significantly lower NSS prevalence than those without remission. The remitters showed a significant reduction in NSS subscores except sensory integration, whereas the non-remitters reported a significant reduction of the overall NES score.	The findings were limited to male patients and might not be able to generalize to female patients. Relatively small sample for the follow-up of NSS at only 2 time points. The association between NSS and negative symptoms was not examined.
Mayoral *et al.*, 2008	Twenty-four patients with first-episode schizophrenia were recruited.	Patients were followed up over a 2-year period.	Thirty healthy controls were recruited and followed up 2 years later.	NES	Patients demonstrated significantly more NSS than controls at baseline assessment, and showed a significant decrease in the sensory integration, others and total NES score over the follow up period, whereas the controls only demonstrated a significant decrease in the total NES score.	Small sample size followed up only at 2 time points. The association between NSS and negative symptoms was not examined. No additional neurocognitive function assessment was obtained from this study.
Cuesta *et al.*, 2012	One hundred patients with medication naïve psychotic disorders	Only 77 patients were followed at baseline, 1 month and 6 months.	Twenty-eight healthy controls	NES	Patients receiving atypical antipsychotics showed significant improvement on the total NES score and most NES subscales except for frontal signs. Clinically meaningful changes on the NES score ranged from 25% to 50%.	The study was mainly a drug trial study testing the efficacy of risperidone, olanzapine, mixed antipsychotics or no medication. The sample was mixed with psychotic disorders other than schizophrenia. The relationship between NSS and negative symptoms was not examined. No additional neurocognitive function was obtained from the study.
Prikryl *et al.*, 2012	Sixty-eight male patients with first-episode schizophrenia were recruited.	Patients were followed up 4 years later.	None	NES	The patients with remission (57% of the original sample) demonstrated a decrease in the sensory integration and sequencing of complex motor acts. For the patients without remission (43%), they showed increase in the total NES score and other item of the NES. A relationship between NSS and negative symptoms was also found.	The findings were limited to male patients and might not be able to generalize to female patients. Relatively small sample for the follow-up of NSS at only 2 time points.
Mayoral M *et al.*, 2012	One hundred first-episode psychosis patients	Fifty-nine patients were followed up 2 years later.	Ninety-eight healthy controls were recruited and only 80 of them were followed up 2 years later.	NES	Patients showed more NSS than controls both at baseline and the 2-year follow-up . However, the patients demonstrated a significant greater reduction of NSS than the healthy controls at follow-up. No significant differences were demonstrated among different diagnostic groups of schizophrenia, bipolar disorders and other psychoses.	No association of NSS and negative symptoms was evaluated. . No additional neurocognitive function assessment was obtained from this study.

NES: Neurological Evaluation Scale (Buchanan & Heinrichs, 1989); SNE: Standard Neurological Examination (Madsen *et al.*, 1999); Heidelberg Scale (Schroder *et al.*, 1992).

**Table 2:** Demographic and clinical information for patients with schizophrenia with and without prominent negative symptoms, and healthy controls.

	**Patients with Schizophrenia**		**Health controls (N = 62)**	**Comparison**
	**Prominent Negative Symptoms (N = 29)**	**Non-Prominent Negative Symptoms (N = 116)**		
Gender (male: female)	20:9	49:67	32:30	*X*^*2*^ = 6.91, *df* = 2, *P* = 0.032
Handed (right: left)	27:2	110:6	60:2	*X*^*2*^ = 0.65, *df* = 2, *P* = 0.724
Age (year)	22.34 (4.06)	21.69 (3.768)	21.16 (1.89)	*F* = 1.27, *df* = 2, *P* = 0.283
Education (year)	11.72 (1.93)	11.94 (2.22)	12.44 (2.18)	*F* = 1.47, *df* = 2, *P* = 0.233
IQ estimates a	96.22 (14.16)	102.93 (14.39)	111.52 (13.01)	*F* = 13.67, *df* = 2, *P* < 0.001
PANSS				
Total score	60.93 (13.91)	42.16 (14.91)		*t* = 6.14, *df* = 143, *P* < 0.001
Negative symptoms	21.79 (5.22)	9.48 (3.51)		*t* = 15.19, *df* = 143, *P* < 0.001
Positive symptoms	12.34 (5.44)	10.98 (5.05)		*t* = 1.28, *df* = 143, *P* = 0.203
General psychopathology	26.79 (6.10)	21.70 (7.79)		*t* = 3.28, *df* = 143, *P* = 0.001
Duration of illness (month)	2.68 (2.10)	3.43 (4.37)		*t = *-0.90, *df* = 143, *P* = 0.372
Simpson-Angus Scale	0.88 (1.59)	0.40 (0.95)		*t* = 1.55, *df* = 143, *P* = 0.132
Barns Akathisia Scale	0.16 (0.45)	0.16 (0.69)		*t* = 0.04, *df* = 143, *P* = 0.972
Abnormal Involuntary Movement Scale	0.00 (0.02)	0.07 (0.46)		*t* = −0.78, *df* = 143, *P* = 0.438

PANSS: Positive and Negative Syndrome Scale; a, Bonferroni post hoc there is no difference between Prominent and Non-Prominent Negative Symptoms groups (P = 0.065).

**Table 3:** Comparison of neurological soft signs and neuropsychological function performances between patients with schizophrenia and healthy controls at baseline assessment.

	**Patients with Schizophrenia**			**Analysis**^**a**^		**Cohen’s d**		
	**Prominent Negative Symptoms (PNS,N = 29)**	**Non-Prominent Negative Symptoms (NPNS, N = 116)**	**Health controls (HC, N = 62)**	**F**	**P**	**PNS vs NPNS**	**PNS vs HC**	NPNS Vs HC
CNI								
Total scores	7.13 (3.17)	5.61 (3.07)	2.76 (2.21)	19.02	<0.001	0.49	1.60	1.07
Motor coordination	3.68 (1.98)	2.64 (1.88)	1.34 (1.28)	13.47	<0.001	0.54	1.40	0.81
Sensory Integration	2.26 (1.50)	1.99 (1.40)	0.74 (1.13)	12.41	<0.001	0.19	1.14	0.98
Disinhibition	1.19 (0.97)	0.98 (0.93)	0.68 (0.65)	3.21	0.042	0.22	0.62	0.37
Neuropsychological functions								
LM immediate score	6.93 (3.77)	8.41 (3.50)	11.19 (3.47)	11.45	<0.001	−0.41	−1.18	−0.80
LM delayed score	4.83 (3.01)	6.56 (3.67)	9.69 (4.08)	13.87	<0.001	−0.52	−1.36	−0.81
VR immediate score	19.66 (3.82)	20.77 (2.51)	21.97 (1.98)	4.23	0.016	−0.34	−0.76	−0.53
VR delayed score	19.62 (4.50)	20.28 (2.74)	21.44 (2.01)	2.29	0.104	−0.18	−0.52	−0.48
LN span correct items	13.96 (2.89)	15.50 (3.25)	17.84 (3.40)	5.85	0.003	−0.50	−1.23	−0.70
LN span longest items	5.46 (0.95)	6.00 (1.09)	6.68 (1.21)	4.04	0.019	−0.53	−1.12	−0.59
Verbal fluency	17.24 (4.97)	18.04 (5.06)	21.63 (5.51)	5.21	0.006	−0.16	−0.84	−0.68
WSCT perseverative errors	3.18 (3.39)	2.68 (3.10)	0.76 (1.40)	2.70	0.069	0.15	0.93	0.80
WSCT category score	4.85 (1.41)	5.15 (1.41)	5.76 (0.88)	6.42	0.002	−0.22	−0.77	−0.52

CNI: Cambridge Neurological Inventory; LM: Logical Memory; VR: Visual Reproduction; LNS: Letter-Number Span Test; PNS: prominent negative symptoms; NPNS: non-prominent negative symptoms; WCST: Wisconsin Card Sorting Test; a, MANCOVA (Multivariate analysis of covariance) gender and IQ as covariates.

**Table 4:** Trajectories of the neurological soft signs and neuropsychological performances of patients with and without prominent negative symptoms.

	**Group**		**Time**		Group*Time	
	**F**	**P**	**F**	**P**	**F**	**P**
PANSS positive	0.10	0.755	133.78	<0.001	0.74	0.391
PANSS general	3.04	0.084	113.71	<0.001	0.01	0.909
CNI total scores	4.72	0.034	41.16	<0.001	0.06	0.807
CNI motor coordination	7.22	0.009	22.38	<0.001	0.87	0.356
CNI sensory integration	0.66	0.42	22.07	<0.001	0.16	0.695
CNI disinhibition	0.10	0.751	2.35	0.129	0.11	0.737
LM immediate score	2.83	0.097	36.68	<0.001	1.91	0.171
LM delayed score	0.97	0.329	56.61	<0.001	0.04	0.839
VR immediate score	0.98	0.325	2.94	0.090	0.60	0.433
VR delayed score	0.48	0.490	2.72	0.102	0.28	0.597
LN correct items	7.88	0.007	0.54	0.462	3.19	0.079
LN longest items	5.33	0.024	0.24	0.627	1.67	0.202
Verbal fluency	0.61	0.437	0.22	0.638	0.17	0.686
WCST perseverative errors	0.34	0.560	28.81	<0.001	0.07	0.789
WCST category	0.10	0.755	16.19	<0.001	0.39	0.533

CNI: Cambridge Neurological Inventory; LM: Logical Memory; PANSS: Positive and Negative Syndrome Scale; VR: Visual Reproduction; LNS: Letter-Number Span Test; WCST: Wisconsin Card Sorting Test.
